# Experiences with legally mandated treatment in patients with schizophrenia: A systematic review of qualitative studies

**DOI:** 10.1192/j.eurpsy.2020.37

**Published:** 2020-05-14

**Authors:** Joanne E. Plahouras, Shobha Mehta, Daniel Z. Buchman, George Foussias, Zafiris J. Daskalakis, Daniel M. Blumberger

**Affiliations:** 1 Institute of Medical Science, Faculty of Medicine, University of Toronto, Toronto, Ontario, Canada; 2 Temerty Centre for Therapeutic Brain Intervention, Centre for Addiction and Mental Health, Toronto, Ontario Canada; 3 Joint Centre for Bioethics, University of Toronto, Toronto, Ontario, Canada; 4 Bioethics Department, Toronto Western Hospital, University Health Network, Toronto, Ontario, Canada; 5 Dalla Lana School of Public Health, University of Toronto, Toronto, Ontario, Canada; 6 Krembil Brain Institute, University Health Network, Toronto, Ontario, Canada; 7 Campbell Family Mental Health Institute, Centre for Addiction and Mental Health, Toronto, Ontario, Canada; 8 Department of Psychiatry, Faculty of Medicine, University of Toronto, Toronto, Ontario, Canada; 9 Schizophrenia Division, Centre for Addiction and Mental Health, Toronto, Ontario, Canada

**Keywords:** involuntary treatment, qualitative, review, schizophrenia

## Abstract

**Background:**

Patients with severe mental illness, including schizophrenia, may be legally mandated to undergo psychiatric treatment. Patients’ experiences in these situations are not well characterized. This systematic review of qualitative studies aims to describe the experiences of patients with schizophrenia and related disorders who have undergone legally mandated treatment.

**Methods:**

Four bibliographic databases were searched: CINAHL Plus (1981–2019), EMBASE (1947–2019), MEDLINE (1946–2019), and PsycINFO (1806–2019). These databases were searched for keywords, text words, and medical subject headings related to schizophrenia, legally mandated treatment and patient experience. The reference lists of included studies and systematic reviews were also investigated. The identified titles and abstracts were reviewed for study inclusion. A thematic analysis was completed for the synthesis of positive and negative aspects of legally mandated treatment.

**Results:**

A total of 4,008 citations were identified. Eighteen studies were included in the final synthesis. For the thematic analysis, results were collated under two broad themes; positive patient experiences and negative patient experiences. Patients were satisfied when their autonomy was respected, and dissatisfied when it was not. Patients often retrospectively recognized that their treatment was beneficial. Furthermore, negative aspects of the treatment included deficits in communication and a lack of information.

**Conclusions:**

Intervention research has historically focused on clinical outcomes and the quantitative aspects of treatment. Thus, this study provides insight into the qualitative aspects of patients’ experiences with legally mandated treatment. Recognizing these opinions and experiences can lead to better attitudes toward treatment for patients with schizophrenia and related psychiatric illnesses.

## Introduction

Schizophrenia is a serious psychiatric illness that affects approximately 1% of the population worldwide [1,2]. It involves emotional, cognitive, and behavioral symptoms [3] that are often difficult to treat [4]. The annual economic burden of schizophrenia is estimated to vary between US$94 million and US$102 billion annually, with indirect costs responsible for between 50% and 85% of total expenses [5].

Patients with severe mental illness, including schizophrenia, may be legally mandated to receive treatment. In the early 20th century most admissions to psychiatric institutions were involuntary, due to stigma, overcrowding and understaffing at the facilities. In industrialized societies, involuntary hospitalization legislation has since undergone various modifications [6,7]. There has been an overall movement toward deinstitutionalization [8].

Despite attempts to standardize legally mandated treatment, rules, and regulations vary regionally and globally [7,9]. For example, within Canada, there exists 12 Mental Health Acts, which equates to almost one separate act per province and territory [10]. Overall, the criteria for involuntary detention in most countries requires that (a) a patient be suffering from a severe mental disorder and (b) compulsory treatment is required to protect the patient or others [9].

Rates of legally mandated admissions for psychiatric patients are increasing [11]. Among this population, patients with schizophrenia are more likely to be involuntarily admitted than patients with other disorders [12–14]. Across the European Union, up to 50% of legally mandated admissions are for schizophrenia and related psychiatric disorders [11,15,16]. Rates of involuntary admission for mental disorders across the European Union vary from 6 per 100,000 people in France to 218 per 100,000 people in Finland [15]. Heterogeneity in the rates of involuntary admission globally can be partially explained by different legal frameworks, individual-, system-, and area- related characteristics [12].

Data regarding the effectiveness of legally mandated treatment is mixed. When compared to voluntary patients, involuntary patients tend to fare better with certain outcomes, and worse with others [17–19]. A systematic review compared outcomes for acute adult psychiatric patients who were admitted involuntarily and voluntarily. Length of stay, risk of readmission and involuntary readmission were at least equal or greater for involuntary individuals. Involuntary patients had higher suicide rates, lower levels of social functioning, and equal levels of general psychopathology and treatment compliance [20].

The literature regarding patients’ attitudes toward their legally mandated psychiatric treatment is limited. One review evaluated patients with psychiatric illness and their positive and negative experiences with involuntary treatment [21]. Areas of importance included patients’ perceived autonomy and participation in decision-making, feelings of being cared for, and their sense of identity [21]. Unlike the previous publication [21], our review includes a larger number of studies and emphasizes the experiences of patients with schizophrenia. Through this systematic review of qualitative studies, we aim to primarily describe the experiences of patients with schizophrenia and related disorders who were legally mandated to undergo psychiatric treatment. By understanding perspectives, healthcare providers can identify methods to strengthen patient–provider relationships [22] and improve compassionate care [23], which may enhance treatment adherence, satisfaction and well-being [23]. Thus, improving patient experience could lead to better clinical outcomes [24,25].

## Methods

### Search strategy

Four electronic bibliographic databases were searched: CINAHL Plus (1981 to May 9, 2019), EMBASE (1947 to May 9, 2019), MEDLINE (1946 to May 9, 2019), and PsycINFO (1806 to May 9, 2019). The databases were searched for key words, text words and medical subject headings (MeSH) related to schizophrenia, legally mandated treatment, and patient experience. Duplicate records were removed. All titles and abstracts identified by the literature search were independently reviewed for study inclusion by two authors (J.E.P., S.M.). Any disagreements were resolved through discussions with a third author (D.M.B.). If the inclusion criteria were unclear from the abstract, the full text was retrieved for further assessment. References within each of the included studies were also searched to identify additional relevant publications. The reference lists of relevant reviews identified using search terms “compulsory treatment,” “mandated treatment” and “involuntary treatment” in the Cochrane Database of Systematic Reviews were searched. Email correspondence with authors was completed to obtain additional study info.

### Inclusion and exclusion criteria

Studies where at least 50% of patients had a diagnosis of schizophrenia or schizoaffective disorder were included. Qualitative studies that reported on the experiences of patients under any form of legally mandated treatment were included. Different forms of legally mandated treatment included involuntary treatment, community treatment orders, community care orders, and forensic patients. Mixed-methods studies were included if qualitative findings were presented separately. Searches were limited to publications in the English language. Case-studies, commentaries, reviews, first-person accounts, and abstracts were excluded.

### Data synthesis and analysis

A thematic analysis [26,27] was completed to synthesize data from each of the included studies. Thematic analysis was selected because it is a flexible approach which can provide a detailed account of data. It is also recommended when researchers want to gain insight into patients’ experiences [28].

We selected the two broad themes of positive and negative patient experiences *a priori.* These included any positive and negative aspects of treatment that patients may have experienced while under any form of legally mandated treatment. We chose to proceed with positive and negative patient experiences to be consistent with a previous review of qualitative studies [21] that identified these overlying themes from the patients’ perspective in their data.

Two authors (J.E.P. and S.M.) independently read all the included studies and extracted themes from the results sections. Where applicable, each sentence from the results section was coded as referring to a positive or negative patient experience.

Next, we went through the lines of coded text to identify subthemes. After discussing within the research team, we came to an agreement on subthemes to be used. The four most commonly identified subthemes were included in this review.

The first reviewer (J.E.P.) has experience completing systematic reviews. Her interpretations are driven by academic interests, rather than clinical experience. The second reviewer (S.M.) has experience working and assessing symptoms in patients with severe mental illness. D.M.B. is a psychiatrist with extensive experience treating patients with schizophrenia and other forms of severe mental illness. People with a lived experience of schizophrenia spectrum illness were not involved in developing or validating the thematic analysis.

### Study quality assessment

The critical appraisal skills program (CASP) was used to assess the quality of each of the publications that met the inclusion criteria [29]. The CASP tool contains 10 questions regarding the clarity, methods, and results of the studies. Studies were accordingly ranked as low (0–3 points), medium (4–7 points), and high quality (8–10 points). Study quality was independently assessed by two authors (J.E.P. and S.M.). Any disagreements were resolved through discussions with a third author (D.M.B.).

## Results

### Search results

**Figure 1.**
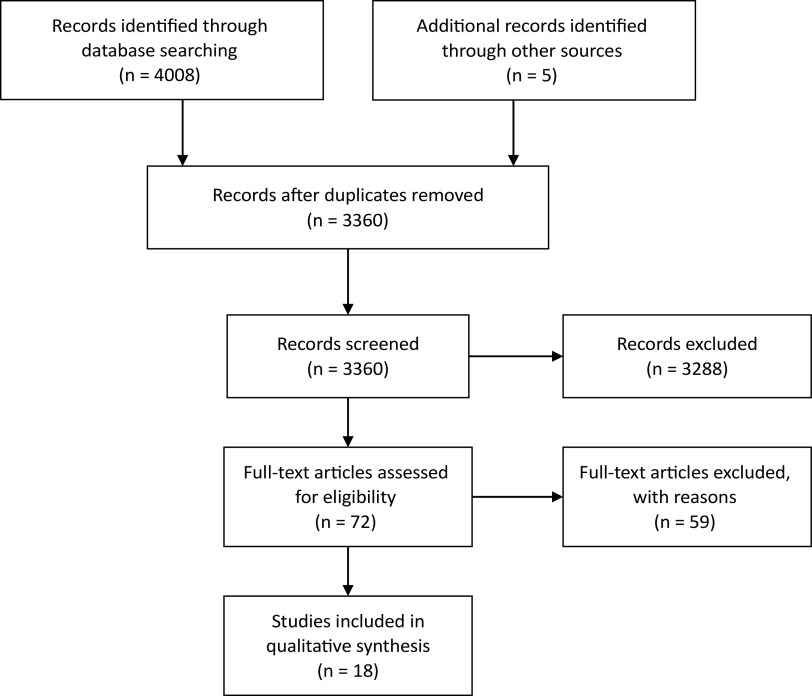
Study flow diagram.

The search completed on May 9, 2019 yielded a total of 4,008 abstracts through electronic searches of MEDLINE (*n* = 586), EMBASE (*n* = 957), CINAHL Plus (*n* = 2102), and PsycINFO (*n* = 363). Searching the reference lists of included studies yielded an additional five citations. A total of 648 duplicate references were removed, and an additional 3,288 references were excluded through the review of titles and abstracts. After assessing 72 full-text articles for study eligibility, an additional 59 references were excluded for failing to meet the inclusion criteria. A total of 18 articles are included in this systematic review of qualitative studies. For detailed search results, see study flow diagram (Figure 1).

### Characteristics of included studies

A total of 18 publications with 401 patients were included in this systematic review of qualitative studies [30–47]. Each study had a clearly stated goal or objective and explored slightly different aspects of patients’ experiences with various forms of legally mandated treatment. Studies were completed in England (*n* = 5), New Zealand (*n* = 3), Australia (*n* = 2), Sweden (*n* = 2), Austria (*n* = 1), Canada (*n* = 1), Ireland (*n* = 1), Japan (*n* = 1), Norway (*n* = 1), and Scotland (*n* = 1). Some of the methodological approaches included thematic analysis, grounded theory, and interpretative phenomenological analysis. All of the included studies, with the exception of one [30] were rated as high quality. Detailed study characteristics can be found in Table 1.

**Table 1.** Detailed study characteristics.

Publication	Objective/purpose	Country	Participant characteristics	Legal status	Study type	Method of analysis	Study quality
Adams and Hafner [30]	To determine the experiences of patients and their relatives with the Guardianship Board, and their attitudes towards Guardianship; and to assess the need for any changes to Guardianship Board procedures	Australia	Total number of participants: 79. Schizophrenia or schizoaffective disorder (*n* = 58, 74%); bipolar disorder (*n* = 10, 13%); organic mental syndrome/disorder (*n* = 8, 10%)	Guardianship	Questionnaire	Not mentioned	Medium
Andreasson and Skarsater [31]	To describe patients’ conceptions and experiences of care in compulsory treatment for acute onset psychosis	Sweden	Total number of participants: 12. Schizophrenia (*n* = 5, 42%); delusional disorder (*n* = 3, 25%); schizoaffective disorder (*n* = 1, 8%); and unspecified nonorganic psychosis (*n* = 3, 25%)	Compulsory admission	Interview	Phenomenographic	High
Atkinson et al. [32]	To evaluate the use of community care orders in the first 33 months of their availability and to assess psychiatrists’ and patients’ views of their usefulness	Scotland	Total number of participants: 45. Schizophrenia (*n* = 35, 78%); bipolar disorder/manic depression (*n* = 10, 9%); schizoaffective disorder (*n* = 3, 7%); learning disability plus another condition (*n* = 2, 4%); schizoaffective disorder vs. manic depression (*n* = 1, 2%). Only 12 (27%) of participants were interviewed	Community care order	Interview	Thematic	High
Brophy and Ring [33]	To offer a voice to both consumers and service providers about their experiences and views of current practice and policy implementation in an area that can have a profound effect on the rights of consumers	Australia	Total number of participants: 30. Participants were most likely to have a diagnosis of schizophrenia	Community treatment order	Interview	Thematic	High
Canvin et al. [34]	To examine participants’ experiences of the mechanisms via which the community treatment order was designed to work: the conditions that form part of the order and the power of recall	England	Total number of participants: 26. Schizophrenia (*n* = 18, 69.2%); bipolar (*n* = 7, 26.9%); and other psychosis (*n* = 1, 3.9%)	Community treatment order	Interview	Grounded theory	High
Fahy et al. [35]	To explore the perspectives of patients subject to supervised community treatment within two mental health teams in Mereyside	England	Total number of participants: 17. Schizophrenia (*n* = 7, 70.6%); schizoaffective (*n* = 3, 17.6%); delusional disorder (*n* = 1, 5.9%); and mental and behavioral disorder secondary to alcohol (*n* = 1, 5.9%)	Supervised community treatment	Interview	Not mentioned	High
Gault [36]	To analyze service-user and carer perspectives on medication compliance and their experience of compulsory treatment	England	Total number of participants:11. Schizophrenia (*n* = 10) and bipolar disorder (*n* = 1)	Compulsory treatment	Interview	Adaptation of grounded theory	High
Gibbs [37]	To consider the impact of community treatment orders of Maori patients and their extended family and the associated views of mental health professionals	New Zealand	Total number of participants: 8. 6 schizophrenia, 1 schizoaffective, 1 bipolar	Community treatment order	Interview	Inductive	High
Gibbs [38]	To explore the views of patients with recent experience of community treatment orders	New Zealand	Total number of participants: 22. Schizophrenia 13 (59%); affective psychosis 3 (14%); and schizoaffective 5 (23%)	Community treatment order	Interview	Inductive	High
Gibbs [39]	To examine the views of service users, family members and mental health professionals about the impact of involuntary outpatient treatment	New Zealand	Total number of participants: 42. 23 (55%) schizophrenia, 10 (24%) affective psychosis, 7 (17%) schizoaffective, 1 (2%) personality disorder, and 1 (2%) other	Community treatment order	Interview	Inductive	High
Johansson and Lundman [40]	To obtain a deeper understanding of involuntarily hospitalized psychiatric patients and their experiences with involuntary hospital care	Sweden	Total number of participants: 5 (>60% schizophrenia)	Involuntarily admission	Interview	Phenomenological hermeneutic	High
Mezey et al. [41]	To explore definitions, experiences, and perceptions of recovery in patients with severe mental illness, currently detained in medium secure psychiatric provision	England	Total number of participants: 10. Paranoid schizophrenia (*n* = 7, 70%) and schizoaffective disorder (*n* = 3, 30%)	Legal detainment	Interview	Thematic	High
Murphy et al. [42]	To explore the experiences of individuals admitted to the hospital involuntarily under the Mental Health Act 2001 in the Republic of Ireland	Ireland	Total number of participants: 50. Nonaffective psychotic disorder (includes schizophrenia, brief psychotic disorder, schizophreniform disorder [*n* = 26, 52%]); affective psychotic disorder (includes bipolar affective disorder and major depressive disorder [*n* = 16, 32%]), alcohol use disorder (*n* = 3, 6%); other (*n* = 2, 4%); no diagnosed disorder (*n* = 2,4%); and no diagnosis available (*n* = 1, 2%)	Involuntary admission	Interview	Inductive	High
Niimura et al. [43]	To elucidate patients’ challenges immediately after hospital discharge following acute psychiatric inpatient care to clarify how to improve inpatient care and postdischarge follow‐ups	Japan	Total number of participants: 18. Schizophrenia spectrum disorder (*n* = 18, 100%)	Involuntary admission	Interview	Inductive	High
Nordberg [44]	To report the experiences of successful graduates of a Canadian Mental Health Court	Canada	Total number of participants: 9. All had been diagnosed with a mental health problem that featured psychosis. The two most common diagnoses were schizophrenia and bipolar disorder	Diversion	Interview	Interpretative phenomenological analysis	High
Riley et al. [45]	To explore (a) patients’ experiences with Outpatient Commitment, and (b) how routines in care and health services affect patients’ everyday living	Norway	Total number of participants: 11. Schizophrenia, schizotypal, and delusional disorders (*n* = 11, 100%)	Outpatient commitment	Interview	Thematic narrative analysis	High
Sibitz et al. [46]	To establish a typology of coercion perspectives and styles of integration into life stories	Austria	Total number of participants: 15. Schizophrenia (*n* = 2), schizoaffective disorder (*n* = 6), bipolar disorder (*n* = 5), acute psychotic disorder (*n* = 1), drug-induced psychosis (*n* = 1)	Involuntary commitment	Interview	Modified grounded theory	High
Stroud et al. [47]	The explore the experiences of service users, practitioners and nearest relatives, to identify key factors and good practice in relation to community treatment orders	England	Total number of participants: 21. Schizophrenia, schizoaffective disorder, and bipolar affective disorder (*n* = 21)	Community treatment order	Interview	Interpretative phenomenological analysis	High

### Thematic analysis of results

In this synthesis, published results from each of the included studies were coded as reporting either positive or negative experiences. Four additional subthemes were identified and classified under the two main themes of positive and negative patient experiences. Further study details can be found in Tables 1 and 2.

**Table 2.** Positive and negative patient experiences.

Publication	Positive (*n* = 14)		Negative (*n* = 15)	
	Independence and autonomy (*n* = 8)	Recognition that treatment/admission was beneficial (*n* = 12)	Restrictions on autonomy, rights, and freedoms (*n* = 12)	Deficiencies in communication/lack of information (*n* = 10)
Adams and Hafner [30]	No	Yes	Yes	Yes
Andreasson and Skarsater [31]	Yes	No	No	Yes
Atkinson et al. [32]	Yes	Yes	Yes	Yes
Brophy and Ring [33]	No	Yes	Yes	Yes
Canvin et al. [34]	Yes	Yes	Yes	No
Fahy et al. [35]	No	No	Yes	Yes
Gault [36]	No	No	Yes	Yes
Gibbs [37]	Yes	Yes	Yes	No
Gibbs [38]	Yes	Yes	Yes	No
Gibbs [39]	Yes	Yes	Yes	No
Johansson and Lundman [40]	Yes	Yes	Yes	Yes
Mezey et al. [41]	No	Yes	No	No
Murphy et al. [42]	No	No	Yes	Yes
Niimura et al. [43]	No	No	No	Yes
Nordberg [44]	No	Yes	No	No
Riley et al. [45]	Yes	No	Yes	No
Sibitz et al. [46]	No	Yes	No	Yes
Stroud et al. [47]	No	Yes	No	No

### Positive patient experiences (*n* = 14)

#### Independence and autonomy (*n* = 8)

Despite being under compulsory treatment, patients valued independence [31]. One patient said; “He [the psychiatrist] tells me where I can live and where I cannot live, he is the one that is in charge of me [but] not in charge of my whole life. I still go to the pubs; he cannot stop me from going to the pubs to see my mates” [39]. Some patients reported that being on a community treatment order (CTO) allowed them to gradually gain more independence before being discharged [38]. Patients enjoyed taking responsibility for their own care [40], and realized that they can obtain “freedom” if they follow the doctor’s orders [47]. Patients reported that community care orders [32], guardianship or supervised discharged orders [34], and community treatment orders [37–39] provided them with greater autonomy than other forms of legally mandated treatment. One patient reported enjoying “More freedom, responsibilities, choices, decision-making of my own rather than being told what to do all the time” [34].

#### Recognition that treatment/admission was beneficial (*n* = 12)

Some patients reported that controls on their behavior, medications, and the electroconvulsive therapy that they received were beneficial. For example, “It placed controls on me that I needed when I first became ill”; “It made me take the tablets when I did not want to and needed to”; “It showed somebody cared”; and “They persuaded me to have the electric-shock treatments which have benefited me enormously” [30]. Patients accepted their illness and the need for medications; “Does not bother me. I realize now that I’ve got to take the pills because I feel too much of a lack of adrenaline if I do not. They calm me down” [32]. Patients believed that involuntary treatment was appropriate to manage those in crisis [33]. Some patients requested to be placed under supervised orders because they found them to be beneficial [34]. Patients expressed that community treatment orders helped them recover from illness, become independent, form close friendships, become aware of their illness, and prevented them from becoming severely ill [37]. A number of patients claimed that being on a CTO prevented suicide or self-harm, and that treatment was needed and ensured their safety [38–40,46,47]. One patient said; “And then, that you maybe for your own safety and the safety of others have to be locked in on the ward… it is done for my own good” [40]. Furthermore, patients believed that treatment was required for recovery [41], and legally mandated treatment was described as a defining life experience [44].

### Negative patient experiences (*n* = 15)

#### Restrictions on autonomy, rights, and freedoms (*n* = 12)

In reference to Guardianship Boards, patients said that it amounted to an infringement on their rights; “The fear of the police intruding on my privacy to take away my freedom was a real disadvantage when I was under a treatment order” [30]. Patients reported that community care orders (CCOs) placed too many restrictions on their life. One individual did not like being told what to do by a “mere slip of the lass” [32]. Several patients compared their involuntary treatment to being placed in jail [33,38]. Involuntary admission negatively affected patient freedom, lifestyle, and privacy; “I cannot do things I want to do. Travel, get a job, things like that” [34]. They experienced a restriction on their autonomy and a fear that they would be detained if they did not obey; “The CTO restricts my liberty. The police can come to my flat whenever they want. They own my life. I’ve got no liberty” [35]. In addition to loss of autonomy, patients reported feelings of coercion, and a recognition that as an involuntary patient, their views were no longer relevant [36]. Furthermore, they felt restricted in relation to their place of residence, physical movement, and social and work opportunities [37]. One patient was unable to visit his supportive father due to restrictions on the distance he could travel from his home [39]. Patients were overwhelmed by rules and inflexibility [40].

#### Deficiencies in communication/lack of information (*n* = 10)

Patients disliked an absence of appropriate communication and information; “I wasn’t told what was going on. It was like a court hearing. They should talk to you more”; and “I would have liked more discussion with the Board about my illness” [30]. Patients would appreciate more communication with healthcare providers to divert their attention away from the negative aspects of their illness; “I think they could talk to me more often. I think it’s good, if you are lost in your own psychotic thoughts, then it’s good to be a little distracted… get something else to think about… It does not have to be about illness. It can be about the weather, sports, or whatever. I think they could do that more. Talk to the patients.” [31]. Patients were confused about the conditions and procedures surrounding their admission [32,33,42]. In one study, only 35% of patients reported satisfaction about the written information provided about their supervised community treatment; “I received info but did not understand it”; “written information was not clear for me to understand”; and “I cannot remember what it said” [35]. Patients would have liked their views to be considered during their admission; “They talk about me behind my back, then they tell me what the team decided, the second time, they did not even have a ward round thing, the nurses just came up and said ‘right you are sectioned again’ I thought What?, it was a bit of a liberty” [36]. Furthermore, patients reported “being outside and not seen or heard”, receiving care without information, receiving a treatment they do not understand, being ignored, and wanting to be involved; “…I felt so extremely bad and I wanted someone to talk to, it was at night I recall. But he said ‘I cannot help you’ he said and he just went away, he could at least sit by my side. Or talk to me about anything then, I’m not, I do not expect him to work miracles but just being there would have been enough…” [40]. Patients would have appreciated more ordinary conversations and reported that healthcare staff often appeared aloof and unavailable [46].

## Discussion

This systematic review of qualitative studies explored the experiences of patients diagnosed with schizophrenia and related disorders while receiving variations of legally mandated treatment. Undergoing legally mandated or involuntary treatment is a complex and multi-faceted process that varies by jurisdiction [48,49]. This review is the largest review conducted to date including 18 qualitative studies with a total of 401 patients. Overall, patients reported satisfaction when their autonomy was respected and dissatisfaction when it was not. Patients retrospectively acknowledged that certain aspects of their treatment were beneficial and led to improved health outcomes. Importantly, patients were dissatisfied when there was limited communication of lack of information provided by healthcare staff.

Autonomy is a key tenet of healthcare ethics and outlines that patients should be permitted to make informed decisions about their healthcare, with freedom from controlling influences [50]. Often patients who are admitted to hospital involuntarily lack capacity to consent to treatment, which limits their autonomy [51]. Patient autonomy is complicated by legally mandated treatment, since the patient’s diagnosis often interferes with their ability to consent to or decline treatment [9,52]. Patients who are more engaged in their treatment decisions exhibit improved treatment outcomes [53–56]. Patient participation includes being involved in decision making or expressing attitudes about different treatment options [57]. An increased emphasis on collaborative care has the potential to increase the participation of patients in their own treatment and improve their autonomy [58].

Many patients described in this review retrospectively acknowledged that their treatment was beneficial. This is consistent with previous research, and is especially true for patients who achieved improvement of symptoms [59,60]. In a previous systematic review, the majority of patients who were admitted involuntarily exhibited substantial improvement with treatment [59]. Furthermore, between 33% and 81% of patients who were admitted involuntarily described their treatment as beneficial and/or justified [59]. It has been argued through paternalistic grounds that involuntary treatment can be justified, namely that overruling of the patient’s autonomy is not always permanent. For example, involuntary treatment during a psychotic episode may restore a patient’s capacity, which would allow them to then make autonomous decisions [61].

Another theme that emerged from this review was that patients disliked deficiencies in communication and a lack of information regarding their treatment. Communication between patients with severe mental illness and their healthcare providers can be challenging [62,63]. However, improved patient communication leads to better health outcomes [64,65]. Patient participation can be enhanced by working on the patient–physician relationship, recognizing the patient’s knowledge about their illness experiences, incorporating patient perspectives into shared decision making, and allocating sufficient time for patient participation [66]. Furthermore, training of healthcare providers to improve communication skills with patients with severe mental illness has been shown to have a positive effect of patient experience in the therapeutic setting [67,68].

We proceeded with a qualitative approach, which allowed us to gain insight into patients’ experiences [69]. Qualitative research seeks to establish a holistic narrative and is flexible in its design [70]. The qualitative research method that is most appropriate to use depends on the purpose of the study [71]. Some of the qualitative approaches used in the reviewed studies include thematic analysis, grounded theory, phenomenology, inductive, and narrative. Each of these methods has its own advantages and disadvantages, which makes a direct comparison challenging. The narrative approach aims to explore the life of a person, phenomenology aims to understand the essence of the experience, and grounded theory develops a theory grounded in data from the field [72]. Thematic analysis develops themes based on the data [73]. Inductive analysis is similar to grounded theory and establishes potential themes *a priori* [74].

The overall trend toward the deinstitutionalization of patients with psychiatric illness has led to an increased use of mandatory treatment in the community [75]. However, evidence regarding the efficacy of treatment in the community for patients with psychiatric illness is mixed [75–77]. There are various reasons why someone may be treated in the community rather than the inpatient setting [78]. Mandatory treatment in the community was originally suggested to prevent frequent readmissions [78]. It was also viewed as a method to increase access to care for patients with psychiatric illness [79–81]. This long-term approach supports the goal of recovery and re-integration into society, where patients are better prepared to pursue their personal education, social, and vocational goals [78,82].

This review provides some insight into patients’ experiences with legally mandated treatment beyond those reported in prior quantitative studies [18]. Elaborating on the experiences of individuals with schizophrenia and related disorders was a recommended area of focus from previous reviews [21,83]. Despite the use of legally mandated treatment across the globe and accepted as a method to protect patients and society, ethical challenges continue to exist [9,84]. Ethical issues related to the involuntary treatment of patients with psychiatric illness include conflicts between the principles of beneficence, autonomy and nonmaleficence. In medical ethics, beneficence (do good), nonmaleficence (do no harm), and autonomy should be valued equally [61,85]. Healthcare providers work toward achieving a balance between patients’ interests and those of society, and patient autonomy can be compromised when addressing this balance [85]. According to healthcare ethics, involuntary treatment is acceptable when it is in the patient’s best interest [86].

There were several limitations of this systematic review. Three of the studies included in this review [37–39] were published by the same authors and used the same patient group. However, each study addressed slightly different aspects of patients’ experiences with community treatment orders. An additional limitation is the coding of qualitative data into positive and negative patient experiences. The analysis positioned findings as within a binary, whereas patient perspectives likely fall along a spectrum. Some aspects of care may be positive, others negative, and formerly positive experiences may at times be negative, and vice versa. Furthermore, there is the potential for selection bias in the included studies. For instance, results may have been coded as “positive” or “negative” because participants may have wanted to contribute socially desirable responses. Patients may have been inclined to provided positive responses, especially if members of their treatment team were running the study. In contrast, some participants may have seen the research as an opportunity to let their care team know how unhappy they were with their treatment, leading to predominantly negative descriptions. Furthermore, many of the included studies recruited patients from Oceania and Europe. There were limited studies published in North America, Asia, Africa, and South America. There are some potential explanations for this geographical imbalance. First, we excluded studies published in a language other than English. Second, rates of legally mandated treatment vary across the globe and are higher in some of the nations that were included in this review. For example, Australia tends to have higher rates of involuntary treatment than other English-speaking nations, such as the United States and Canada [87]. Furthermore, most specialty mental health services in Australia are delivered in community settings and one-sixth of services comprise involuntary treatment [87]. Third, while completing this review, it appeared that there was more research in general across Europe related to legally mandated treatment [88]. This may be partially explained by the finding that higher rates of involuntary hospitalization are associated with a lower rate of absolute poverty, with higher gross domestic product and healthcare spending per capita, an increased proportion of foreign-born people in a population, and greater amounts of inpatient beds [89]. Finally, criteria, procedures [7,90], and rates [11] of treatment in patients with psychiatric illness vary globally.

When patients with schizophrenia and related disorders are legally mandated to undergo treatment, they can have both positive and negative experiences. Retrospectively, many patients recognized that their treatment was beneficial, however, efforts should be made towards improving patient autonomy and ensuring clear communication with patients about their illness and treatment. Improving patient experiences is critical, as rates of involuntary admission are increasing and people with schizophrenia and related disorders are at higher likelihood of receiving legally mandated treatment [91–94]. Training for healthcare providers that encourage patient-centered care may have positive effects on patient health behavior and health status [95]. Findings from this study on patients’ experience could better inform healthcare providers when treating this vulnerable group of patients.
